# The biennial cycle of respiratory syncytial virus outbreaks in Croatia

**DOI:** 10.1186/1743-422X-5-18

**Published:** 2008-01-28

**Authors:** Gordana Mlinaric-Galinovic, Robert C Welliver, Tatjana Vilibic-Cavlek, Suncanica Ljubin-Sternak, Vladimir Drazenovic, Ivana Galinovic, Vlatka Tomic

**Affiliations:** 1 Department of Virology, Croatian National Institute of Public Health, Rockefellerova 12, 10000 Zagreb, Croatia; 2 Division of Infectious Diseases, Department of Pediatrics, Children's Hospital, State University of New York at Buffalo, 219 Bryant Street, Buffalo, NY 14222, USA

## Abstract

The paper analyses the epidemic pattern of respiratory syncytial virus (RSV) outbreaks in children in Croatia. Over a period of 11 consecutive winter seasons (1994–2005) 3,435 inpatients from Zagreb County aged from infancy to 10 years who were hospitalised with acute respiratory tract infections were tested for RSV-infection. RSV was identified in nasopharyngeal secretions of patients by virus isolation in cell culture and by detection of viral antigen with monoclonal antibodies.

In the Zagreb area, RSV outbreaks were proven to vary in a two-year cycle, which was repeated every 23–25 months. This biennial cycle comprised one larger and one smaller season. Climate factors correlated significantly with the number of RSV cases identified only in the large seasons, which suggests that the biennial cycle is likely to continue regardless of meteorological conditions. Knowledge of this biennial pattern should be useful in predicting the onset of RSV outbreaks in Croatia, and would facilitate planning for the prevention and control of RSV infections in the region.

## Findings

Respiratory syncytial virus (RSV) frequently causes acute respiratory tract infections (ARTI) among children. In investigations of the epidemiology of viral respiratory infections in Croatian children over four seasons in the 1980s, RSV was determined to be the agent of 20–34% of inpatient ARTI [1,2]. Our study of RSV-genotypes circulating in Zagreb and Vienna from 1987–1994 showed that they were similar to the pattern of expression of these genotypes globally [3].

In temperate climates, RSV infections occur in winter and in the early spring [1,4]. The role of climate in causing this epidemic pattern has not been evaluated in the area including and surrounding Croatia. We set out to examine the timing of RSV epidemics and the relationship of various meteorological factors and the number of RSV infections in children over 11 consecutive years in Zagreb County. The county covers an area of 3,719.355 km^2^, and includes a population of 1,088,841 inhabitants in the northwest part of Croatia.

This study was conducted as a part of the scientific project #0005002 approved by the Ethic Committee of Croatian National Institute of Public Health (CNIPH). The study period lasted from 1 July 1994 to 1 July 2005. This retrospective cohort study comprised 3,435 inpatients with an ARTI from Zagreb County who were 0 to 10 years of age (median = 7.5 months).

Samples of nasopharyngeal secretions collected from each patient were transported at +4°C to the Department of Virology, CNIPH within 24 hours of collection. The samples were processed immediately on receipt for rapid detection of RSV, adenovirus, influenza virus (type A and B) and parainfluenza virus (type 1–3) by direct fluorescence assay (DFA, Light Diagnostics, Chemicon International, Inc., Temecula, CA) and for isolation of these viruses in cell culture (Hep-2, HeLa, MDCK). Influenza-positive (DFA-detection) samples were inoculated in MDCK line. After development of the cytopathic effect typical for RSV in Hep-2 cell culture, DFA was then applied to cell culture to confirm RSV infection. Detection of influenza and parainfluenza viruses in cell culture was completed using haemadsorption with guinea pig erythrocytes. Serotyping of influenza and parainfluenza (type 2 and 3) isolates was accomplished using DFA. Adenoviral typing was done by neutralization with hyperimmune sera (Central Public Health Laboratory, London).

Data regarding climate for the area (Zagreb-Maksimir, #920-08/06-01/228) including air temperature and relative humidity were obtained from the Department for Climatology, Croatian Meteorological and Hydrological Service, Gric 3, 10 000 Zagreb. The Zagreb County climate has four distinct seasons. The average temperature in winter is 1°C (34°F) and, in summer, 20°C (68°F) [5].

### Statistical analysis

Pearson coefficient of correlation and non-parametric Mann-Whitney U-test were performed using STATISTICA for Windows, StatSoft, Inc. (1999), Tulsa, OK, USA as appropriate. Differences with a probability of p < 0.05 were considered to be significant.

Among the 3,455 subjects studied, RSV was detected in 32.2%, adenovirus in 3.9%, parainfluenza in 3.7%, influenza in 2.9%, and combined detections of RSV and another virus in 0.4%. The mean age of recruited patients was 13.4 months (SD = 18.7) and was similar in the virus negative and all the virus positive groups reported.

**Figure 1:**
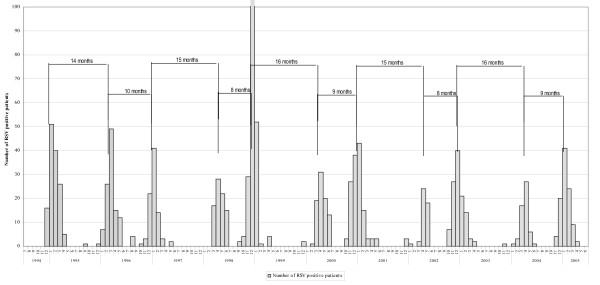
Seasonal occurrence of respiratory syncytial virus infections (number of cases) in Croatia (1994–2005).

**Figure 2:**
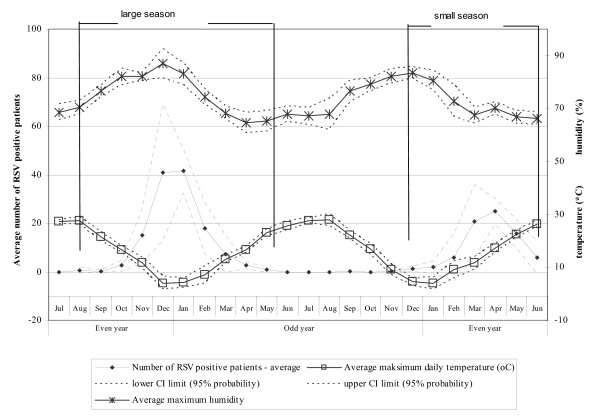
Average values of number of RSV-positive patients, maximum daily temperature and maximum humidity with 95 percent probability confidence interval of biennial cycle of RSV-infections in Croatia.

An analysis of the monthly occurrence of RSV outbreaks through the 11 years of the study established an alternating cycle. Thus RSV epidemics peaked in December/January of years 1994/95, 1996/97, 1998/99, 2000/01, 2002/03, and 2004/05 ("large seasons"), but in March/April of years 1996, 1998, 2000, 2002, and 2004 ("small seasons") (Fig. 1). This finding suggests that there are two separate seasons (Fig. 2) because July, August and September of even years have, on average, only 0.0, 0.7 and 0.3 infections, respectively.

In the average large season there were 130 RSV-positive patients, while the average small season had only 77 RSV-positive cases (p = 0.018, Mann-Whitney U-test). There were no differences in the age of RSV-positive patients between small and large seasons (Mann-Whitney U-test; p = 0.496). The mean age of these subjects in small seasons was 13.1 ± 18.5 months and, in large seasons, 13.8 ± 19.0 months.

The two-year cycle, repeated every 23–25 months, is divided into two subperiods (measured between peaks of seasons): the prolonged subperiod lasts for 14–16 months, the short one for 8–10 months (Fig. 1). Thus, after the appearance of a major RSV epidemic, a minor one follows 14–16 months later, and then another major epidemic 8–10 months later.

In large seasons the number of RSV cases was inversely related to the average maximum daily temperature (Pearson correlation coefficient; r = -0.7; p < 0.001) and directly to average maximum humidity (Pearson correlation coefficient; r = 0.6; p < 0.001) (Fig. 2). In small seasons, however, the number of RSV cases was not significantly correlated with temperature (r = 0.06; p = 0.64) and was inversely correlated to relative humidity (r = -0.3; p < 0.01). In large seasons, in months with an average maximum temperature over 25°C there are virtually no RSV infections, whereas RSV cases were detected most often in months with the maximum daily temperature of around 5°C (Fig. 2).

The peak of epidemic activity of RSV varies in differing geographic areas. In temperate climates, RSV activity increases in the winter months, but RSV may occur year-round in equatorial areas. A study conducted in Greece demonstrated that the peak of the RSV epidemic occurred in February, with the season beginning in November and ending in May [6]. Another study done in Italy showed the epidemic peak occurred in February in one season, and in March during another season [7]. A Tunisian study also found the incidence of RSV infections to peak in winter months [8]. Our study demonstrated that RSV outbreaks in Croatia have a biennial cycle similar to that reported for Germany [4], but unlike the monophasic annual cycle reported from the United Kingdom [9].

The relationship of RSV activity to meteorological conditions has not been studied extensively, particularly in the area near Croatia. The air temperature was the factor most closely correlated with the number of documented cases of RSV in Croatia in large seasons, suggesting that low temperature plays a more important role than humidity in these seasons. Similar results were reported by Wang TL, et al. [10]. Nevertheless, in our study, temperature was not significantly related to the number of RSV cases that could be documented in smaller seasons.

Our study demonstrated that higher air humidity is associated with a higher number of RSV-positive patients in large seasons, which is in the opposite of findings of Lapena et al [11]. A possible explanation of this discrepancy exists in the findings of a study of climate on RSV activity in regions varying widely in geography and climate [12]. In this study a certain range of humidity (50–65%) appeared to support optimal survival of RSV, with reduced activity of RSV above or below this range. This identification of an optimal humidity is based on a survey carried out in 9 distinct geographic regions in different hemispheres with widely varying humidity [12], so the observed effect of humidity is probably quite general. Therefore in areas with particularly dry summers, winter peaks of RSV will correlate directly with greater humidity in winter. Negative correlations may be expected between winter peaks of RSV activity and humidity in areas with wet summers.

We suspect that using hospitalisation of children as a marker of community activity impairs our statistical analysis. In large epidemic seasons, RSV activity (actually, severe disease in infants) is related to climate. In years following major epidemics (small seasons), we suspect that persisting immunity in infants and young children infected the previous year reduces – not the total number of RSV cases in the community, necessarily – but rather the spread of infection from partially immune older children to infants whom we monitor for infection. That is, the number of infants hospitalised may not increase until the community epidemic has persisted longer, even though the magnitude of the epidemic in older individuals may be relatively similar between years. Statistical associations of RSV activity and climate are also generally limited by the possibility that most transmission of RSV may occur indoors, and would therefore be less related to climate.

Our findings of a repeated biennial RSV cycle and the influence of climate on RSV activity add to previous information generated largely in the western hemisphere. Importantly, using our present findings (Fig. 1) and the late peak of the 2005/06 RSV epidemic, we correctly predicted that the next RSV outbreak in Croatia should peak in December 2006/January 2007. These annual predictions may be useful prospectively in planning the institution of measures to control RSV infection. These would include determining appropriate hospital staffing, the timing of cohorting of infants hospitalised for respiratory illness, and the use of prophylactic and therapeutic antiviral products.

## Authors' contributions

GMC made substantial contributions to conception and design, analysis and interpretation of data; involved in drafting the manuscript, final approval of the version. RCW made substantial contributions to conception and design, involved in revising the manuscript critically; final approval of the version. TVC made substantial contributions to acquisition of data, analysis and interpretation of data; involved in drafting the manuscript. SLS made substantial contributions to acquisition of data, analysis and interpretation of data; involved in drafting the manuscript. VD made substantial contributions to acquisition of data. IG made substantial contributions to acquisition of data, analysis of data; involved in drafting the manuscript. VT made substantial contributions to acquisition of data, analysis of data; involved in drafting the manuscript. All authors read and approved the final manuscript.

## Consent

Written informed consent was obtained from the patient for publication of this work. A copy of the written consent is available for review by the Editor-in-Chief of this journal.
